# Consequences of emotional stimuli: age differences on pure and mixed blocks of the emotional Stroop

**DOI:** 10.1186/1744-9081-5-14

**Published:** 2009-03-02

**Authors:** Victoria Ashley, Diane Swick

**Affiliations:** 1 Research Service, Veterans Affairs Northern California Health Care System, Martinez, California, USA; 2 Department of Neurology, University of California at Davis, Davis, California, USA

## Abstract

**Background:**

Studies of aging and emotion suggest that older adults show diminished responsiveness to negative information, possibly resulting from increased emotion regulation, but the mechanisms accounting for this effect are uncertain.

**Methods:**

To examine whether aging affects the allocation of attention to negative stimuli, we compared 20 younger and 20 older adults on 2 versions of the emotional Stroop task: "pure blocks," in which all words in each block were either emotional or neutral, and "mixed blocks," a pseudorandomized design in which either a negative emotional or a neutral category word was always followed by six neutral words. The emotional Stroop task typically elicits slower reaction times for naming the font color of negative emotional words compared to neutral, but no studies have examined the effects of aging on the immediate and sustained components of the emotional Stroop effect.

**Results:**

Both groups showed an emotional Stroop effect on pure blocks manifest as slower RTs on the emotional, relative to the neutral, block. However, only younger adults showed persistent slowing that carried over from emotional words onto subsequent neutral words in mixed blocks.

**Conclusion:**

These results suggest that the consequences of emotional stimuli may differ with age. Younger and older adults showed equivalent interference from the emotional words themselves, but older adults did not show a sustained effect of negative information.

## Background

Aging is often associated with a more optimistic outlook on life. For example, survey data from 72 countries, and mental health measures from Europe, both illustrate that psychological well-being and happiness increase with age, after reaching a nadir between 40–50 years [1]. What contributes to this increase in happiness? One factor could be that the "negativity bias," a robust increased salience for negative information in attention and memory seen in younger adults [2-5], is decreased or nonexistent in older adults [6-9]. Older adults have shown tendencies to regulate negative emotional stimuli using methods such as avoidance, inhibition, or disengagement [10-13]. However the effective use of these techniques also depends on the resource demands involved in the task [14]. Additionally, performance on tasks that tap more automatic processes involving negative stimuli, such as threat detection and affective priming, is intact [11,15]. Thus, both task and individual variables are relevant to age-related changes in the processing of negative information.

Little is known about the mechanism(s) for the relative decrease in the saliency of negative information with aging. Kisley, Wood & Burrows [6] state, "it is unclear whether the bias reversal arises from an age-related increase in responding to positive information or an age-related decrease in responding to negative information."(p. 838) Indeed, studies also indicate that older adults exhibit a "positivity effect," or a relative preference for positive information in attention and memory with age [16-19] [but see [20] for conflicting results]. Socioemotional selectivity theory (SST), a motivational account for the positivity effect, proposes an attentional shift toward positive information to maximize emotional goals as lifetime limits approach [16]. The ability to regulate emotion – i.e., to disengage from or avoid negative emotional stimuli – may be necessary to decrease the salience of negative stimuli.

To examine age-related changes in the processing of negative information, we chose the emotional Stroop task, a variant of the classic Stroop task, which involves the need to disengage from distracting affective stimuli [21]. Although the effects of the classic Stroop and the emotional Stroop appear similar – a slowing in response times – these tasks engage different mechanisms of interference [22-24]. While the classic Stroop creates a response conflict between an incongruent color and word (i.e., the word 'RED' in font color blue), color is incidental to the emotional Stroop, which involves only emotional and neutral stimuli.

The emotional Stroop has been extensively utilized in clinical populations in which words related to an area of concern for an individual (i.e., snakes or spiders for phobics) will elicit slower response times than neutral or even other emotional words [25]. However, studies involving healthy populations are fewer, often specific in scope, and less consistent in their findings [22,26]. For healthy individuals, the emotional Stroop effect is typically observed as a significant slowing of reaction times (RTs) on "pure blocks" (or 'blocked presentations') of emotional words, relative to blocks of neutral words. The effect is less often found with blocks that are mixtures of emotional and neutral words, or "mixed blocks." Pure blocks are reported to elicit larger disruption effects than mixed [27,28]. Additionally, the emotional Stroop task has been described as a task which indexes automatic processes that capture attention, creating a bias toward threat material [25]. But studies of the task using meta-analyses [21] and comparisons of different block types [22] have concluded that the more relevant process may involve a difficulty in disengagement from the negative stimuli, or a carry-over slowing effect, rather than an early automatic capture of attention.

Initial evidence for carry-over slowing in the emotional Stroop was shown in a 1986 emotional Stroop study using blocked presentations by McKenna, in which he described what he called 'an emotional lingering effect' occurring beyond the presentation of the emotional word itself, characterized as interference or slowing on the neutral block of words presented after the emotional block [29]. In two later emotional Stroop studies, Waters, Sayette and Wertz (2003) [30] and Waters, Sayette, Franken, & Schwartz (2005) [31] also found a carry-over slowing effect using a mixed format of the emotional Stroop task with concern-related words for smokers and heroin addicts and on a mixed stress Stroop in healthy normals. Their findings lent support to the generalizability of the carry-over effect across a range of domains.

To more closely examine the relative contributions of the different interference effects in the emotional Stroop, McKenna and Sharma (2004) [22] developed a pseudo-randomized mixed block paradigm in which emotional words appeared in a fixed sequence across time at a rate of once for every 6 neutral words. This structure allowed the distracting effect of the emotional word to be examined across several adjacent neutral words (subjects did not report awareness of the sequence). Their results suggested two types of interference effects, a 'fast' (within trial) effect and a 'slow' (between trials) effect, with the contribution of the fast effect found to be small, and the contribution of the slow effect found to be strong, consistent, and appearing as a carry-over effect on the trial immediately following the negative emotional word.

To our knowledge, only one published study [26] has examined age-related differences in emotional interference effects using the emotional Stroop, and that study used a randomized block design. Wurm et al. [26] included the factors of both arousal and valence in their emotional Stroop design, reasoning that high arousal words would engage automatic activations of word meanings, and so would be more likely to create a Stroop effect. They predicted that older adults would be more vulnerable to interference effects due to the reductions in cognitive resources with aging that could limit their ability to ignore distractions. As predicted, older but not younger adults showed greater interference from high arousal words. But interestingly, neither group displayed an emotional Stroop effect for negative valence words relative to neutral or positive. That outcome may have been due to the randomly mixed block type, in which interference effects are more difficult to detect [27,28]. The use of the mixed block design in this case makes the evaluation of the emotional Stroop effect across age difficult to determine. Additionally, the use of the randomized format did not allow for a further investigation of the mechanisms for interference across time within blocks.

Wurm et al.'s [26] focus on cognitive resource limitations of aging, however, is relevant, since studies have shown age-related declines in cognitive control and resource limitation-related reductions in the ability to effectively inhibit distracters when engaging in emotion modulation [17]. A recent divided attention study by Knight et al. [14] investigated cognitive control and selective visual attention in older and younger adults. They found that when available cognitive resources were overwhelmed in the divided attention condition, older adults seemed to lose the ability to successfully engage motivational goals. Although older adults were less attentive to negative stimuli in the full attention condition, they could not avoid the negative stimuli in the divided attention condition, and the outcome reversed: older adults attended more to negative stimuli when they were distracted [15].

However, while older adults may be more vulnerable to cognitive resource limitations, they are also more likely to engage top-down regulation toward motivational goals when emotional information is involved [17], as predicted by the socioemotional selectivity theory (SST) and studies indicating that emotion regulation is enhanced [32] or maintained [33] with age. The question of the relative contributions of task type and difficulty, availability of cognitive resources, and motivational goals is complex and unlikely to be answered in a single study. While the emotional Stroop task cannot address emotional motivations – since the overt directions in the task require ignoring word content and inhibiting an emotional response – the use of different block types in the emotional Stroop provides a means to consider the relevance of presentation method on distracting emotional information across age groups.

In the present study, RT and error data were collected on two types of blocks of an emotional Stroop task for older and younger adults, pure and mixed, with the mixed block following a similar design to McKenna and Sharma's [22] pseudo-randomized mixed block design (Exps 3 & 4). While a pure block format is more likely to produce an emotional Stroop effect, it also conflates the 'fast' and 'slow' effects described by McKenna and Sharma, and so does not allow for an understanding of either effect individually. Thus we chose to use both the pure and mixed formats. Because our study differed from the McKenna and Sharma design in notable respects – we used a fixed order of blocks for all subjects (they used a Latin Square design), voice-onset RTs (theirs was button-press), and a much longer inter-stimulus interval of 1500 ms (theirs was 32 ms) – we did not expect to replicate their results. However, the pseudo-randomized design of mixed blocks by McKenna and Sharma would allow for close study of any 'carry-over' effects between trials to help determine where differences might arise after exposure to a negative word.

If the negativity bias in older adults is decreased or nonexistent relative to younger adults, and if this bias can be attenuated, even when attentional resources are limited, older adults may be engaging a motivational strategy to avoid, inhibit or disengage from the distracting effects of negative words. If this were the case, we would expect older adults to show less interference overall – less RT slowing – for negative relative to neutral words than younger adults. Although Wurm et al. [26] predicted arousal effects in their study, they did not predict or find emotional Stroop valence effects for negative words relative to neutral or positive. Our study used only high-arousal negative words versus neutral and did not use a randomly mixed format, making it methodologically different from the Wurm et al. study and more likely to detect interference slowing.

Based on previous emotional Stroop research comparing pure and mixed block designs [21,22,27], we expected to see interference effects for both groups on pure blocks, but more so for younger adults, due to their larger negativity bias relative to older adults (negative words would slow their RT to name the colors of words more than older adults). Mixed blocks are less likely to produce emotional Stoop interference overall, so we predicted weaker interference effects – less RT slowing – on mixed blocks relative to pure blocks, across groups. Additionally, the pseudo-randomized order of words in the mixed blocks allows the slow carry-over effect to be examined across adjacent neutral words. If older adults do disengage more effectively from negative stimuli than younger adults, we would expect them to show a shorter duration or smaller effect of interference – less RT slowing – that carries-over onto adjacent neutral words in mixed blocks.

## Methods

### Subjects

Participants were 20 older and 20 younger healthy, right-handed (1 left-handed) adults with equal numbers from each gender. Younger adults ranged in age from 18 to 31 years (*M *= 25.2 yrs; *SD *= 3.9 yrs; Male *M *= 26.0 yrs, Female *M *= 24.4 yrs) and older adults ranged in age from 62 to 80 years (*M *= 70.0 yrs; *SD *= 5.73 yrs; Male *M *= 70.0 yrs, Female *M *= 70.0 yrs). Participants were recruited via notices on the Internet, ads in a local paper, and fliers placed around the community. All participants were paid and signed informed consent statements approved by the Institutional Review Boards of the Veterans Affairs Medical Center and UC Davis. Exclusion criteria included drug or alcohol use, psychotropic medication use, head injury, and any psychological or neurological disorder. One subject was excluded due to uncertainty about prescription medication use for depression. This study was part of a larger study that also included adults with prefrontal cortex lesions, which will be reported separately.

### Stimuli

Stimuli were colored words shown one at a time in the center of a computer screen, either neutral, neutral category (school-related), or emotional (negative). Each word was 48 pt size, in the colors red, blue, green or yellow and displayed in Times font on a black background. Words were presented in the center of the screen at a distance of approximately 56 cm from the viewer. Neutral, neutral category and emotional words were matched for number of letters and frequency using Francis & Kucera [34] (Mean number of letters, frequencies: Pure, emo = 5.92, 37.54; Pure, neu = 5.96, 36.96; Mixed, emo = 5.08, 11.71, Mixed, neu category = 5.04, 18.58; Mixed, neu = 5.08, 11.71) and later confirmed for frequency matching using the English Lexicon Project database [35] to ensure the validity of the measure [36]. Arousal ratings for emotional words were determined according to Affective Norms for English Words (ANEW) [37]. For emotional words, only high arousal negative valence words were used (i.e., horror, bomb, panic).

Words were presented in two types of blocks: pure blocks and mixed blocks. In pure blocks, all words in each block were either neutral or emotional. In mixed blocks, a pseudo-randomized design was used which repeated a pattern of 7 word types, in which the word in Position 1 was an emotional word in emotional mixed blocks, or a neutral school-related category word in neutral mixed blocks, while all other words (words in Positions 2–7) were neutral words.

#### Pure blocks

Pure blocks (blocks 1 & 2) had an inter-stimulus interval (ISI) of 3500 ms with words displayed for 500 ms. Pure blocks consisted of 24 emotional and 24 neutral words, each randomly repeated three times for a total of 72 words in each block. Block 1 was the neutral pure block, consisting of all neutral words, and block 2 was the emotional pure block, consisting of all emotional words.

#### Mixed blocks

Mixed blocks (blocks 3–6) were presented in a fixed order that alternated between neutral and emotional mixed blocks. Trials had an ISI of 1500 ms (words displayed for 500 ms). Thus, mixed block trials occurred faster than pure block trials, but the words were displayed for the same amount of time. Some studies have not shown any emotional Stroop effects in mixed formats [27,28], although the pseudo-randomized design on which this study is based, [22] did show an emotional Stroop effect. Many emotional Stroop designs use a small number of emotional words and repeat them. In order to avoid habituation effects in our study, our pure block words only repeated 3 times, and none of our mixed block words in Position 1 were ever repeated – each mixed block consisted of 24 unique emotional or neutral category words in Position 1 (12 per block), and 288 neutral words (72 per block). Additionally, a few neutral buffer words (2–4 words) were placed at the start of each mixed block to help ensure that subjects were unaware of the repeating pattern. No subject reported awareness of the pattern.

Three types of words were used in the study: emotional category words, neutral category words, and neutral words. Emotional category words consisted of negative high-arousal words from Affective Norms for English Words [37]. In the original study by McKenna and Sharma [22], in order to eliminate the possibility that the repeating sequences of words (1–7) might be more salient or surprising because of the categorical nature of the emotional stimuli, a neutral semantically-related category word type (transport-related words, i.e., airplane, ferry, bus) was created to match emotional words in word position 1. In our study we used school-related words (i.e., locker, quiz, teach) because these were better matched to the emotional words for part of speech. Neutral words were used in Positions 2–7 for both emotional and neutral mixed blocks. Only words in Position 1 were emotional or neutral category words. Each emotional mixed block had only negative emotional words in Position 1, and each neutral mixed block had only neutral category words (school-related words) in Position 1. As with pure blocks, the order of mixed blocks was fixed: *neutral-emotional-neutral-emotional*.

### Procedure

Participants viewed a total of 6 blocks of words, two pure blocks followed by four mixed blocks. Each word was shown one at a time on a computer screen in a dimly lit and sound attenuated room. Participants were instructed to say the color of the word into a microphone and to ignore what the words said. They began with a short practice block. Reaction times were recorded with a voice-onset triggered microphone.

## Results

Only correct responses were included in results analyses (average percentage of error RTs removed: YAs = 1.68%; OAs = 2.74%). Behavioral exclusion criteria included participants with more than 25% error rates (see [26]), which did not apply to any of our participants. Additionally, to decrease variance, trial data were trimmed such that RTs longer than 2 SDs above the subject's block mean were removed [38,39] (average percentage removed: YAs = 5.51%; OAs = 5.75%), and RTs faster than 200 ms (false triggers due to vocal artifacts such as stuttering or coughs) were removed (average removed: YAs = 0.31%; OAs = 0.76%). Although an upper limit of 3000 ms was also used, no responses reached this limit.

### Pure blocks

Reaction time and error results for pure blocks were analysed in repeated measures 2 × 2 ANOVAs with Valence (neutral, emotional) as the within-subjects factor, and Group (YAs, OAs) as the between-subjects factor. Additional ANOVAs included gender as a factor, however, no significant gender differences were observed.

#### RTs

**Figure 1:**
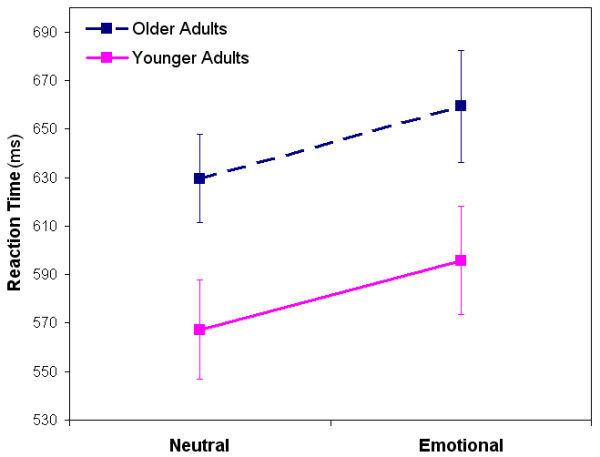
**Reaction times for older and younger adults on pure blocks**. Both groups were significantly slower on emotional compared to neutral words.

Results indicated a significant main effect of Group [*F*(1,38) = 4.71, *p *= 0.036], with overall RTs for older adults slower than younger adults (Means: OAs = 644.55 ms, YAs = 581.52 ms). A significant main effect was also shown for Valence [*F*(1,38) = 20.77, *p *< 0.0001], with both groups slower on emotional relative to neutral words, confirming the emotional Stroop effect (Fig. 1). However, no interaction effect was shown for Valence × Group [*F*(1,38) = 0.009, *p *= 0.924], suggesting that aside from older adults being slower overall, no differences in behavior were shown between groups on pure blocks of the emotional Stroop. Studies show that older adults will often be slower than younger adults on both cognitive [40,41] and emotional tasks [42].

#### Accuracy

An analysis for accuracy on pure blocks showed a non-significant trend for an interaction effect of Valence × Group [*F*(1,38) = 3.57, *p *= 0.067]. But since performance was at ceiling for both groups (Means, emo, neu: OAs = 98.5%, 97.6%; YAs = 98.8%, 99.3%) and comparisons of neutral and emotional block accuracy scores within each group showed no difference across valence (YAs: [*t*(1,19) = 1.45, *p *= 0.162], OAs: [*t*(1,19) = 1.39, *p *= 0.180]), it is unlikely that the trend reflected any meaningful difference.

### Mixed blocks

RT and error results for mixed blocks were analysed in 2 × 2 × 7 ANOVAs, with Group (YAs, OAs) as the between-subjects factor, and Valence (neutral, emotional) and Word Position (1,2,3,4,5,6,7) as the within-subjects factors. The Tukey-Kramer test for multiple comparisons was used on mixed block comparisons.

#### RTs

**Figure 2:**
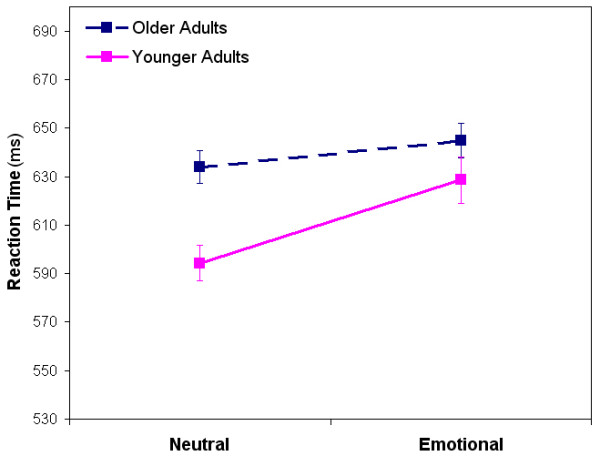
**Reaction times for older and younger adults on mixed blocks**. Younger adults were significantly slower on segments of the mixed blocks involving emotional words than those involving neutral category words. Older adults showed only a trend for similar slowing. Overall mixed group reaction times were not significantly different.

Unlike pure blocks, the mixed blocks showed no main effect of Group [*F*(1,38) = 0.966, *p *= 0.332]. The three-way interaction of Group × Valence × Position was not significant [*F*(6,228) = 0.59, *p *= 0.738], but an interaction effect of Group × Valence was shown [*F*(1,38) = 4.43, *p *= 0.042] (Fig. 2) indicating that younger, but not older adults, were significantly slower on emotional mixed blocks than neutral mixed blocks.

Although the interaction effect for Group × Position was not significant [*F*(6, 228) = 0.896, *p *= 0.498], a significant interaction across groups for the factors of Valence × Position was indicated [*F*(6,228) = 6.27, *p *= 0.0001]. Separate ANOVAs conducted for each group for Valence × Position revealed that younger adults were significantly slower on some word positions in emotional relative to neutral mixed blocks [*F*(1,19) = 12.87, *p *= 0.002], while older adults showed only a trend for such slowing [*F*(1,19) = 3.91, *p *= 0.063]. *A priori *paired t-tests for each group showed that younger adults were significantly slower on all but one word position in emotional relative to neutral blocks (*p *< 0.023), while older adults were only slower on words in Position 1 (*p *= 0.009) and Position 7 (*p *= 0.011) (Table 1). Thus, emotional words had a greater effect on subsequent responses to neutral words for younger than for older adults.

**Table 1:** Mixed block reaction times by word position

Word position							
	1	2	3	4	5	6	7
**Older Adults**							
Neutral	625.12*	638.70	643.16	630.92	637.59	633.91	627.80*
Emotional	649.68*	639.10	640.51	639.68	647.98	645.12	652.07*
**Younger Adults**							
Neutral	586.04*	599.06*	597.66	596.72*	595.93*	597.63*	586.78*
Emotional	638.33*	632.31*	616.23	625.52*	633.90*	621.27*	632.56*

*Note: *All words are neutral except words in position 1. Emotional word 1 is a negative emotional word and neutral word 1 is a neutral school-related category word, such as, *locker, quiz, teach*.

(* = Significant differences between neutral and emotional words, *P *< 0.05)

Because mixed blocks were pseudo-randomly mixed, with words in Position 1 being either emotional or neutral category, and words in Positions 2–7 being all neutral, we were interested in examining if RT slowing would be evident at the emotional word, after the emotional word, or as an overall effect. Thus, we conducted separate ANOVAs for words in Position 1, and averages of words in Positions 2–7.

An ANOVA for words in Position 1 showed no main effect of Group [*F*(1,38) = 0.706, *p *= 0.406], nor a significant interaction of Valence × Group [*F*(1,38) = 2.82, *p *= 0.101], but did show a main effect of Valence [*F*(1,38) = 22.24, *p *= 0.0001]. This result suggests that age did not influence the fast component of the emotional Stroop effect for Position 1 words in mixed blocks. Further comparisons for each group confirmed significant RT slowing for negative emotional words compared to neutral category words in Position 1 (YAs: [*t*(1,19) = 3.75, *p *= 0.001]; OAs: [*t*(1,19) = 2.91, *p *= 0.009]).

**Figure 3:**
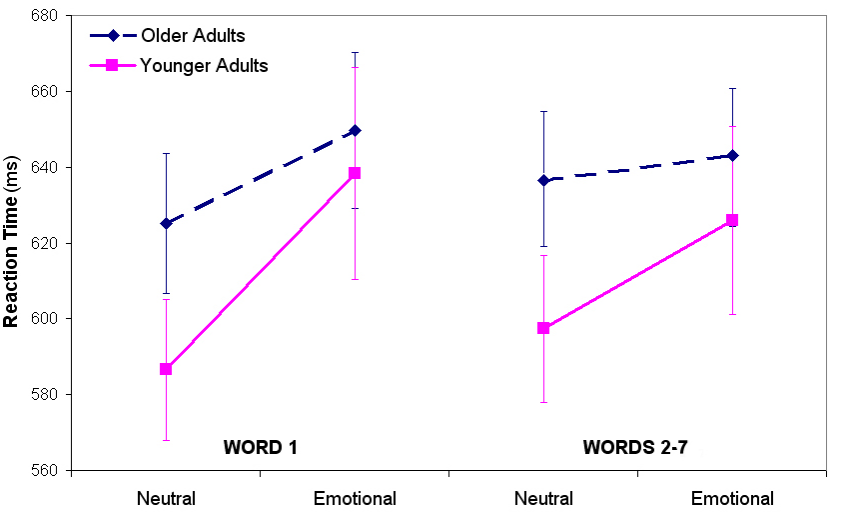
**Reaction times for older and younger adults on Position 1 words (emotional or neutral category words) versus Position 2–7 words (neutral words following Position 1 words)**. Both groups showed significant slowing on emotional Position 1 words. Younger adults were also significantly slower on Position 2–7 words of emotional mixed blocks, while older adults were not.

An ANOVA for words in Position 2–7 showed no main effect of Group [*F*(1,38) = 0.990, *p *= 0.326], but did show a main effect for Valence [*F*(1,38) = 10.56, *p *= 0.0024] (Fig. 3), and an interaction effect of Group × Valence [*F*(1,38) = 4.74, *p *= 0.036], suggesting that both groups were slower on words 2–7 in negative mixed blocks relative to neutral, but also, that such slowing did differ between the groups. Comparisons revealed that younger but not older adults were significantly slower for words in Positions 2–7 of negative emotional compared to neutral mixed blocks (YAs: [*t*(1,19) = 3.43, *p *= 0.003]; OAs: [*t*(1,19) = 1.65, *p *= 0.116]). This difference between the groups indicates that the slowing effects of the emotional words carried over onto neutral words in negative emotional mixed blocks for younger adults, but not older adults.

#### Accuracy

An ANOVA conducted for mixed block accuracy scores showed no significant effects (*p *> .1). An analysis of accuracy for words in Position 1 suggested that both groups were less accurate on negative rather than neutral category words [*F*(1,38) = 5.11, *p *= 0.03], however comparisons did not support significant accuracy differences between emotional and neutral words in either group (*p *> .1). Additionally, no significant differences were shown between groups in accuracy for words in Positions 2–7 [*F*(1,38) = 1.95, *p *= 0.171].

## Discussion

The main purpose of the present study was to examine age-related differences in the "negativity bias" – an increased salience for negative information in attention and memory – using pure and mixed block types of an emotional Stroop task. Results indicated that both age groups were significantly slower to report the color of negative emotional words in pure blocks and on negative emotional words in Position 1 of emotional mixed blocks. Furthermore, younger but not older adults displayed carry-over slowing effects in mixed blocks, consistent with other findings of a diminishment of the negativity bias in older adults [6,7]. Our mixed block results suggest that the consequences of negative emotional stimuli may be of shorter duration for older adults.

An important consideration for designing an emotional Stroop experiment is whether a Stroop effect is shown (a significantly slower response time to emotional or concern-related words relative to neutral) to confirm the ability of the measure to detect differences between affective and neutral stimuli. In our study, while older adults were slower overall at color naming in pure blocks, both groups did show an emotional Stroop effect in pure blocks and for words in Position 1 of mixed blocks. However, contrary to our expectations, no Valence × Group interaction was shown in pure blocks. This result did not support our original prediction that older adults would show less interference overall than younger adults. Instead, older adults performed similarly to younger adults on pure blocks.

The only other study to examine the emotional Stroop effect in healthy older and younger adults found no valence differences between positive and negative words, and no emotional Stroop effect at all for younger adults [26]. However, differences were observed for words rated as high, relative to low, arousal. That study employed a mixed block design in which arousal and valence factors were randomized (as noted earlier, emotional Stroop studies using mixed blocks are less likely to find slowing effects than pure block designs). Based on their results, that older adults appeared to be more "prone to automatic activation of the high arousal words," the authors suggest that arousal may be a more relevant factor than valence for older adults, due to its potentially greater costs to emotional and physical health.

Since our study used only high-arousal negative words, the factor of arousal cannot be compared between the studies, but our results on the factor of valence were contrary to Wurm et al.'s observations – if older adults were more prone to high arousal activation, one might have expected to see increased interference for older adults from the high arousal negative words in our study. We did not see this result in either pure or mixed blocks.

At the same time, we were surprised that no valence interaction between groups in pure blocks was evident. According to our prediction, older adults should have shown a smaller relative RT difference between negative and neutral words than younger adults. Instead, both groups performed similarly, despite older adults being slower overall. One possible explanation could be that since negative emotional words appeared consecutively in the pure block condition (mixed blocks display one negative emotional word for each 6 neutral words), cognitive resource deficits might have limited the ability of older adults to effectively disengage from, or avoid, the negative words, or to engage in other strategies they might tend to use to cope with negative information. Mather and colleagues suggest that successful attempts at regulating emotional information may depend on both cognitive resources and the nature of the task at hand, and that older adults who are better on tasks of cognitive control may be more likely to show positivity effects in memory[17]

Could cognitive resource limitations of older adults have played a role in our results, perhaps hampering efforts to regulate the effects of negative words? We conducted correlational analyses of participants' years of education, RTs, and error scores, and did not find any significant relationships on either pure or mixed emotional blocks, neutral or emotional. (YAs: p > 0.4; OAs: p > 0.3). Additionally, older adults' performance on neutral blocks was similar to younger adults, indicating that cognitive resources were not overwhelmed by the basic aspects of the task – the naming of the colors of words shown for 500 ms each over a series of trials. Whether the emotional content of the negative words could have overwhelmed cognitive resources, remains unknown. The inclusion of further tests to determine the limits of the cognitive resources of older adults participating in the study would help to disentangle resources limitations from motivational processes.

Perhaps the nature of the "fast" emotional Stroop effect (the RT slowing on emotional word itself) is age-invariant, while the "slow" emotional Stroop effect (the RT slowing after the emotional word) is not. For example, age-related differences seen in later stages of other information processing tasks, such as memory retrieval, have led to proposals that older adults may engage in a compensatory strategy shift to attenuate performance deficits via controlled processes [43]. A more comprehensive examination of the relationship between age-related differences in early and late stage processing, and age-related differences in the "fast" and "slow" emotional Stroop effects, is needed.

Given that both groups behaved similarly on fast and slow components of pure blocks but differed on mixed blocks, the question remains as to what could account for these group differences. One possibility is that because mixed blocks always occurred after pure blocks, perhaps the repetition of negative stimuli interacted with age, such that over time, the repeated presentation of negative words may have reduced the negativity bias in older adults, relative to younger adults.

To see if our results could have been due to repetition effects, we examined both block types across time, comparing the first third versus the last third of pure blocks, and blocks 1& 2 versus 3 & 4, of mixed blocks (Note that words repeated 3 times in pure blocks, so the analysis provides an indication of true repetition effects. In contrast, no words were repeated across mixed blocks, so the analysis is a measure of habituation, rather than a true repetition effect). If repetition effects differed between groups over time, we expected to see group interactions occur in the factors of Valence × Time × Group. Analyses of RTs for pure blocks [*F*(1,38) = 1.09, *p *= 0.302] and mixed blocks [*F*(1,38) = 0.044, *p *= 0.834] did not indicate any significant interactions, suggesting that the repetition of stimuli over time did not appear to play a role in results. Further ANOVAs across time for words in Position 1 of mixed blocks [*F*(1,38) = 2.25, *p *= 0.142], and for words in Position 2–7 [*F*(1,38) = 0.012, *p *= 0.913], also did not indicate significant interaction effects. These results suggested that the age groups did not significantly differ in repetition effects across time.

Interpreted in terms of the socioemotional selectivity theory, a diminished carry-over effect of negative stimuli for older adults could be the outcome of a motivational goal toward optimising emotional experiences as the limits of life approach, a relative favoring of positive information in attention and memory with age. However, since the emotional Stroop task does not explicitly engage subjects in the processing of emotional material – only color naming – it is unclear whether our results could be related to the motivational goals of emotional regulation. Two recent ERP studies using the emotional Stroop did find, however, electrophysiological evidence that emotional word content is differentially processed, despite instructions to ignore word meanings and only name colors [44,45]. But the question of whether our findings of age differences in the negativity effect could be due to effortful processing to optimise emotional experiences remains unknown.

Several limitations of our study should be noted. First, to ensure identical testing conditions and reduce affective carryover effects between blocks, our study used a fixed order of blocks for all subjects with neutral stimuli before negative for pure blocks. While a fixed order of blocks can introduce practice effects [46], emotional stimuli or even questionnaires can also contaminate or prime later neutral stimuli [47]. Witthöft, Rist & Bailer [48] used a fixed order in their blocked emotional Stroop design (also neutral words first) in order to "avoid an artificial boosting of the emotional intrusion effect." Consequently, they were able to avoid potential affective carry-over effects and clarify a key finding about generalized slowing for participants with elevated health anxiety, even on neutral words, across time. A study that examined order effects directly in an emotional Stroop task assessing inter-generational trauma, however, reported no order effects, but included caveats about the small and unequal group sizes used [49]. In our study the only potential practice effect we noticed was the tendency of older adults to make more errors on the first block (neutral pure block), but that effect did not reach significance.

Second, our study used negative and neutral but not positive words, leaving the effect of positive words unknown. Negative emotional words typically elicit enhanced processing, thus it is not uncommon for emotional Stroop tasks to involve only negative (or concern-related) words versus neutral words [44,50-52]. Additionally, few studies show differences for positive stimuli on the emotional Stroop: in a study of the role of lexical characteristics of words in 32 emotional Stroop studies, Larsen, Mercer & Balota's [36] first analysis ('Behavioral Differences Associated With Word Valence') found virtually no mean naming RT differences between neutral and positive words, but slowing for negative words (neu: 672 ms; pos: 675 ms; neg: 685 ms). Neither emotional Stroop study by McKenna and Sharma [22,53] found RT differences between positive and neutral stimuli. Similarly, in a presentation of the results of an emotional Stroop study comparing 36 older and 36 younger adults at the Cognitive Aging Conference in Atlanta (2006) [54], Osborne and Burke also found no RT differences between positive and neutral words. Finally, the only published emotional Stroop study that compared older and younger age groups [26] reported no differences between positive and negatively-valenced stimuli. Thus, because differences between negative and neutral stimuli have a stronger basis in the literature and a potential role in the negativity bias, we chose to focus on negative versus neutral words. Follow-up studies using positive stimuli are important for future studies of age differences in the emotional Stroop, particularly toward an understanding of the positivity effect.

Finally, because inter-stimulus intervals were longer on pure than mixed blocks (3500 and 1500 ms, respectively, all words displayed for 500 ms), some questions remain as to whether differences between block types may be due to block content, ISI differences, or both. It is known that shorter ISIs on classic Stroop tasks can increase a focus on the irrelevant dimension, while longer ISIs can eventually eliminate the classic Stroop effect altogether [55]. Hence, one might expect that if ISI played a role in results, the (longer ISI) pure blocks may have shown reduced interference, or the (faster ISI) mixed blocks may have shown increased interference effects. Thus, one possible outcome – if mixed blocks had been kept at the longer ISI of pure blocks – is that mixed blocks may not have shown any interference effects. Currently, the uncertainty about the role of ISI differences in this study precludes a determination that block differences, are in fact, due to block type, exclusive of other factors.

The possibility that the lack of a carry-over effect of emotional words onto adjacent neutral words in older adults may have been due to working memory deficits known to occur with aging [56,57] was considered. For example, if older adults failed to maintain a representation of the words, slowing effects could be lessened. However, older adults performed similarly to younger on neutral category words and neutral words in mixed blocks of our study, suggesting that memory deficits on neutral blocks was not an issue.

Kisley, Wood & Burrows [6] provide an interesting summary of recent research which is perhaps useful toward establishing a framework for future studies in this area. They found that neural reactivity to negative images declines linearly with age, but that neural reactivity to positive images is age invariant. Several recent imaging and electrophysiology studies also support a finding that older adults show reduced physiological responsiveness to negative stimuli, even as responsiveness to positive information, financial gains, or self-reported positive affect, may not differ across age [7-9]. In their paper, Kisley, Wood & Burrows cite several points: (1) the intentional suppression of emotional responses to unpleasant stimuli can decrease electrophysiological responses [58], (2) such suppression efforts may originate in prefrontal cortex [17], and (3) the conclusions of Williams et al. [59] also support the idea that, "as people age, they devote more resources to controlling negative emotional responses, but allow automatic responses to positive stimuli to proceed without restraint."(p. 842) Kisley, Wood & Burrows state that the socioemotional selectivity theory behind the positivity effect may "explain some, but not all, findings from the current study."(p. 842)

The emotional Stroop, as presented in McKenna and Sharma's [22] pseudo-randomised design, engages the need to inhibit the distracting influence of negative emotional words in a format observable across time, thus allowing for the disentangling of age-related early and later stage processing differences already seen in other areas of cognition. Using this paradigm, our study – the first to compare the emotional Stroop effect in healthy older and younger adults using pure and mixed block types – found an interesting diminishment of the negativity bias for older adults, but only in the more subtle mixed block format, suggesting that age-related differences to negative information may depend on task conditions and may occur in later rather than early stages of information processing.

## Conclusion

In conclusion, older adults showed a reduction in the lasting effects of negatively-valenced words intermixed with a series of neutral words. We observed an age-related diminishment in carry-over slowing effects for the mixed blocks of an emotional Stroop task, consistent with other findings of a decrease in the "negativity bias" with age. This could be due to the enhanced emotion regulation of negative information by older adults in controlled processing. Older and younger groups showed comparable levels of interference in the emotional Stroop task, manifest as slower RTs for negative emotional words in pure blocks and negative emotional words in Position 1 of mixed blocks. However, older adults did not display the additional carry-over slowing from the emotional words onto adjacent neutral words in mixed blocks that was observed in the young, suggesting that the consequences of negative emotional stimuli may be of shorter duration for older adults. Contrary to our expectations, however, no interaction effect for valence was indicated in pure blocks between groups. Instead, older adults were simply slower overall than younger adults in pure blocks. This is the first study to compare the emotional Stroop effect in healthy older and younger adults using pure and mixed block types. Future studies of age-related differences on the emotional Stroop should include measures of cognitive resource limitations to effectively address the relevance of age-related differences in cognitive resources for this task.

## Competing interests

The authors declare that they have no competing interests.

## Authors' contributions

VA and DS designed the experiment. VA collected and analyzed the data and drafted initial manuscript versions. VA and DS read and approved the final manuscript.
